# Performance and perception on front-of-package nutritional labeling models in Brazil

**DOI:** 10.11606/s1518-8787.2021055002395

**Published:** 2021-04-23

**Authors:** Luisete Moraes Bandeira, Jéssica Pedroso, Natacha Toral, Muriel Bauermann Gubert

**Affiliations:** I Universidade de Brasília Faculdade de Ciências da Saúde BrasíliaDF Brasil Universidade de Brasília. Faculdade de Ciências da Saúde. Programa de Pós-Graduação em Saúde Coletiva. Asa Norte, Campus Universitário Darcy Ribeiro, Brasília, DF, Brasil; Campus Universitário Darcy Ribeiro BrasíliaDF Brasil; II Faculdade de Ciências da Saúde BrasíliaDF Brasil Faculdade de Ciências da Saúde. Programa de Pós-Graduação em Nutrição Humana. Brasília, DF, Brasil

**Keywords:** Front-Of-Package Nutritional Labeling, Nutritional Labeling, Food Labeling, Warning, Nutrition Policy, Health Promotion

## Abstract

**OBJECTIVE:**

To evaluate the performance and perception of five models of front-of-package nutrition labeling (FOPNL) among Brazilian consumers.

**METHODS:**

Cross-sectional study, which applied an online questionnaire to 2,400 individuals, allocated randomly into six study groups: a control group and five others exposed to FOPNL (octagon, triangle, circle, magnifier and traffic light), applied to nine products. We evaluated the understanding of nutritional content, the perception of healthiness, the purchase intention and the perception of Brazilian consumers on the models.

**RESULTS:**

All FOPNL models increased the understanding of the nutritional content and reduced the perception of healthiness and purchase intention, when compared with the control group (41.3%). FOPNL warning models — octagon (62.4%), triangle (61.9%) and circle (61.8%) — performed significantly better than the traffic light (55.0%) regarding the understanding of the nutritional content. The performance of the magnifier (59.5%) was similar to the other four tested models, including the traffic light (55.0%), for understanding nutritional content. The individual analysis of the products suggests a better performance of warnings in relation to the magnifier and the traffic light for the perception of healthiness and purchase intention. Consumers were favorable to the presence of FOPNL, perceiving it as reliable to increase the understanding to nutritional information.

**CONCLUSION:**

FOPNL must be implemented on food labels in Brazil, considering that it increases the nutritional understanding, reduces the perception of healthiness and the purchase intention of products with critical nutrients. Warnings showed a better performance when compared with other models.

## INTRODUCTION

Front-of-package nutritional labeling (FOPNL) is internationally recommended^1^ as a tool to assist the consumer in interpreting quantitative nutrient statements in foods, which are generally difficult to understand and arranged in small print on the back of the package^2^. Almost half of the Brazilian population has difficulty interpreting nutritional information on food labels^3^. By not understanding the content of the products, judgment regarding healthiness and, consequently, the purchase decision of the individual are affected^4^^,^^5^.

Several countries adopt different FOPNL models to help the consumer in this interpretation. Warning models (octagon, circle and triangle), inform, in a simple and direct way, if the product has a high content of some nutrient (sugars, fats, sodium). They have been more efficient in increasing understanding, and consequently, reducing the perception of healthiness and the intention to buy product, when compared with the nutritional traffic light, which informs the low, medium and high content of nutrients, or the Guideline Daily Amounts (GDA), which indicates the percentage of nutrients present in the product in relation to the recommended daily value^6^^–^^10^. In recent years, four Latin American countries — Chile, Peru, Uruguay and Mexico — have adopted the octagon-shaped warning FOPNL as mandatory^11^^–^^14^.

In Brazil, the National Health Surveillance Agency (ANVISA) approved, in 2020, a FOPNL model in a black rectangular format with a magnifier, similar to what has been discussed in Canada^15^^,^^16^. However, only two studies evaluated the performance of this FOPNL model, being inferior to the octagon and the triangle in reducing the time for identification of nutrients in excess among Brazilian adults^9^. The magnifier model was also inferior to the octagon, circle and triangle in increasing the understanding of nutritional content among adults from the United States, Canada, Australia and the United Kingdom^17^.

The performance of the FOPNL models can also be influenced by factors such as motivation for health, ease of preparation and price^18^, as well as by aspects related to the model's own design, such as its ability and draw attention, the ease of the consumer to identify and process their information, familiarity with the FOPNL and the perception of risk generated by it^8^^,^^19^^–^^21^.

Because of this, it is important to conduct local studies to identify the most appropriate FOPNL model for the population of each country^22^. There is a need for studies comparing the performance of different FOPNL models in Brazil, including the magnifier, investigated in only one of the two studies conducted with Brazilian adults that compared more than one FOPNL model^9^^,^^10^.

Therefore, the objective of this study was to evaluate the performance of five models of front-of-package nutritional labeling (octagon, triangle, circle, magnifier and traffic light) in increasing the understanding of nutritional content, reducing the perception of healthiness and the intention to buy product, in addition to identifying the perception of Brazilian adult consumers about these models and the importance of factors related to food choice.

## METHODS

### Study participants

A cross-sectional study was conducted with a sample of 2,400 individuals randomly assigned to six study groups. The sample was made by quotas, being representative of the Brazilian population in relation to sex, economic class and the five macro-regions of the country. The recruitment of participants was carried out digitally, by a company specialized in online surveys that has a register of respondents. Invitations were sent only to people who met the quota profile pre-determined in the sample. As quotas were finalized, invitations were sent to the remaining quotas.

The questionnaire was applied in August 2019. All individuals agreed to their participation by signing the informed consent form. The study was approved by the Ethics Committee in Human Research of the Faculdade de Ciências da Saúde of the Universidade de Brasília (protocol 67420817.7.0000.0030).

### Sample Allocation in Research Groups

The participants were randomly allocated into six groups, 400 of which were in the control group (GC), while the others in one of the five exposure groups: 1) magnifier (n = 400); 2) circle (N = 400); 3) octagon (N = 400), 4) triangle (N = 400) and 5) and nutritional traffic light (N = 400) (Figure 1). The inclusion of a control group allowed the comparison of the performance of the FOPNL models among themselves and the performance of each individually in relation to the absence of FOPNL in the product.

**Figure:**
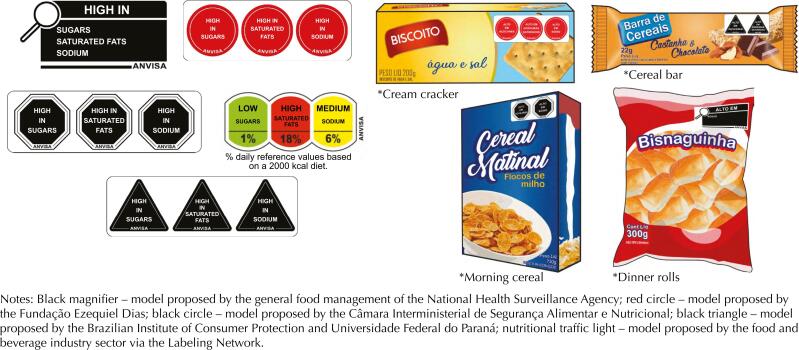
Tested front-of-package nutritional labeling models and examples of product images visualized by participants. Brazil, 2019.

### Position and size of FOPNL models

The FOPNL models (Figure 1) were applied in the upper right corner, in different percentages of the area of the main panel of the product: 15% if the product had high content of sugars, sodium and saturated fat; 10% when high in two of these nutrients; and 5% in one of these nutrients. The magnifier always used 10% of the area.

The magnifier, octagon and triangle were shown in black and the circle in red color. The nutritional traffic light was shown in red, yellow and green colors, indicating respectively the high, medium or low levels of nutrients.

For the definition of low, medium and high nutrient content (free sugars, saturated fat and sodium), the most restrictive nutritional profile model proposed by ANVISA was adopted^23^.

### Product selection

A panel of experts selected nine products commonly consumed by the Brazilian population^24^ and usually perceived as healthy, despite having a high content of at least one nutrient (free sugars, fat and sodium) (Table 1). As performed in a previous study^9^, product images were prepared by a company specialized in graphic design exclusively for this research and did not contain health claims, trademarks or trade names, seeking to neutralize the influence of these factors on the performance of the models (Figure 1).

**Table 1:** Nutritional composition of the products included in the study and content (low, medium and high) of nutrients associated with chronic non-communicable diseases, according to the criteria established in the preliminary report of regulatory impact analysis on nutritional labeling. General Food Management. National Health Surveillance Agency, 2018.

Product	Size of the serving (g/mL)	Nutrient content per serving				Classification of nutrient content		
		Calories (Kcal)	Free sugars (g)	Saturated fats (g)	Sodium (mg)	Free sugars (g)	Saturated fats	Sodium
Requeijão	30	81	7	4.1	144	Medium	High	High
Tomato sauce	60	30	5.8	0	311	Low	Low	High
Sliced bread	50	133	20	0.5	219	Low	Low	High
Cream cracker	30	129	20	1.7	210	High	High	High
Cereal bar	22	91	16	1.6	17	High	High	Low
Morning Cereal	30	111	25	0	147	High	Low	High
Nectar (juice)	200	40	10	0	0	High	Low	Low
Corn	130	129	25	0	606	Low	Low	High
Dinner rolls	40	124	22	0.8	189	Medium	Medium	High

### Data collection

The questionnaire was organized into three sections: 1) characteristics of participants, 2) performance of FOPNL models and 3) consumer perception of FOPNL models.

In Section 1, the characteristics of the participants (sex, age group, education, income and region of housing) were identified, including the importance of factors related to food choice. Ten items were elaborated based on the Food Choice Questionnaire^18^, for which the participants evaluated the importance with answer options on a 5-point Likert scale, ranging from: 1 – “not at all important” to 5 – “very important.” The items were “*I choose food*”: a) easier to prepare; B) that the place of purchase is close to me; c) for the price; d) that are healthier; E) that make me cheerful, relaxed, active/awake; F) natural, without additives or artificial/industrialized ingredients; g) with few calories, sugars or fats; h) from the brand I always usually buy; i) or similar to what I ate in childhood; j) that do not harm the environment, preferring organic foods and avoiding foods with pesticides.

In Section 2, we evaluated the performance of the FOPNL models in increasing the understanding of nutritional content and in reducing the perception of healthiness and the intention to purchase the products shown. Individuals saw, individually and randomly, the nine products with the FOPNL model according to their randomization group. No individual was exposed to more than one type of FOPNL. While viewing each product, participants answered three questions. The first question measured the understanding of the nutritional content of the product: “*in your opinion, does this* product *contain nutrients at higher levels than recommended for a healthy diet?*”. For the purposes of standardization and comparison between the FOPNL models studied, we chose to only keep this question for the traffic light, since this was the only FOPNL model that allowed to quantify medium and low levels of nutrients^9^^,^^10^. The answer options were multiple choices: “too much sugar,” “too much sodium,” “too much saturated fat” or “does not contain any nutrients in too much quantity,” and the participant could choose more than one answer option. Two other questions measured the purchase intention and the perception of healthiness of the products, with answer options on a 5-point Likert scale: “*would you buy this* product?” (1 – “I certainly would not buy it” to 5 – “I certainly would buy it”); and “*you consider this* product”: (1 – “not healthy” to 5 – “very healthy”). The control group visualized the same product, however, without any FOPNL model. If desired, the subjects of all groups could look at the nutritional information table and the list of ingredients of each product by clicking on a button located just below the image of the product.

In Section 3, participants' perception of FOPNL models was evaluated in relation to “ease of identification,” “reliability,” “information processing” and “preference.” These dimensions were based on the acceptability structure proposed by Nielsen^25^, already used in studies on FOPNL^19^^,^^20^. The questions were shown while the participant viewed the FOPNL model of their randomization group in isolation. To assess the ease of identification, the individual answered the following questions/statements: “1. *Did you see this label on the* product you evaluated? (yes or no);” 2. *It was difficult to see this label on the package*,” “ 3. *I found the nutritional information more quickly with this label*.” For “reliability,” the statement was “4. *I trusted the information of this label*.” When evaluating the processing of information, the following statements were shown “5. *I understood the nutritional information more quickly with this label*,” – “6. *I understood this label*,” “ 7. *I felt uncomfortable with this label*.” The following statement allowed assessing the preference: “8. *I would like to find this label on food packaging*.”

Also in this section, we investigated whether the FOPNL models induced the participants to the basic emotion fear^25^ with the statement: “9. *the presence of this label made me afraid*.” Questions 2 to 9 had 5-point Likert scale answer options ranging from 1 – “I totally disagree” to 5 – “I totally agree.” This section was shown only to the participants of the exhibition groups.

### Statistical Analysis

To estimate the sample size, we considered a 95% confidence level, a maximum acceptable error of 2 percentage points, alpha of 0.05 and test power of 95%. The estimation of the sample considered a 57.6% mean number of correct answers for understanding the nutritional content for the traffic light, 79.9% for the triangle^10^, and the use of the one-way ANOVA test, estimating an effect size of 11. Thus, this study should include at least 210 adults per group, to which were added 100% to cover possible data loss or inconsistency, estimating a sample of 2,400 individuals. The G*Power 3.1.9.2 software performed the calculations^2^.

To understand the FOPNL, the percentage of correct items for each product was first calculated according to the participant's response in relation to the presence or absence of the nutrient in excess. For all nine products, we considered the percentage of the participant's correct answers in relation to all products. Subsequently, the means of the percentage of right answers of the exposure and control groups were compared.

We also estimated, for the six groups, the mean of purchase intention and perception of healthiness of the participants in relation to the nine products together and individually. A percentage expressed the visualization of the FOPNL model. We also calculated the means of agreement, according to the Likert scale, considered as a continuous variable, for the questions that evaluated the participants' perception in relation to the FOPNL models.

Pearson's chi-square test (categorical variables) or one-way ANOVA with Tukey's post-test (continuous variables) were used to verify whether there were differences between the groups regarding the characteristics of the participants, the performance of the FOPNL models and the perception of the participants in relation to the FOPNL models between the groups. We considered a 95% confidence interval. All analyses were conducted using the Statistical Package for the Social Sciences (SPSS) software, version 23.0.

## RESULTS

Most of the sample of 2,400 adults was aged between 18 and 34 years (55.1%), 51.2% were women and 37.1% had completed high school. The characteristics of the participants showed no statistical difference between the six research groups.

The mean importance of factors related to food choice (ease of preparation, proximity to the place of purchase, price, preference for healthier foods, with natural content, for weight control and ethical concern in food choice) attributed by the participants were similar between the control and exposure groups (Table 2).

**Table 2:** Sociodemographic characteristics of the study participants and mean of importance attributed to factors related to food choice. Brazil, 2019.

Characteristics		Total sample n = 2,400 (%)	Magnifier n = 400 (%)	Circle n = 400 (%)	Octagon n = 400 (%)	Traffic light n = 400 (%)	Triangle n = 400 (%)	Control n = 400 (%)	p
Gender								
	Male	48.8	48.8	48.0	47.8	49.5	49.5	49.3	0.993^a^
	Female	51.2	51.3	52.0	52.3	50.5	50.5	50.8	
Age group,									
	18–34	55.1	52.8	54.8	56.5	55.5	53.0	58.0	
	35–54	35.9	37.8	35.5	35.5	35.5	37.0	34.0	0.940^a^
	≥ 55	9.0	9.5	9.8	8.0	9.0	10.0	8.0	
Education, in years								
	< 9	3.3	3.3	4.8	3.5	2.0	3.8	2.3	
	9 < 12	10.8	9.5	12.0	9.8	11.8	10.0	11.8	0.510^a^
	≥ 12	86.0	87.3	83.3	86.8	86.3	86.3	86.0	
Income, in minimum wage^b^									
	< 2	48.4	45.8	47.5	48.8	49.0	49.8	49.5	
	2 < 10	46.1	48.3	46.0	48.3	45.3	43.8	45.3	0.569^a^
	≥ 10	5.5	6.0	6.5	3.0	5.8	6.5	5.3	
Region									
	North	5.1	4.5	5.25	5.5	4.75	5.25	5.75	
	Midwest	8.3	8.5	7.5	9	8.5	9	7.75	
	Northeast	23	24.5	23	21.8	22.3	24.5	22.5	1,000^a^
	Southeast	47.8	46.5	48	48.3	50.2	45.5	48.5	
	South	15.5	16	16.25	15.5	14.25	15.75	15.5	
Importance of factors related to food choice — “*I choose food*”:									
Easier to prepare^c^			3.69	3.69	3.51	3.67	3.67	3.80	0.049^d^
That the place of purchase is close to me^c^			3.86	3.66	3.83	3.80	3.83	3.77	0.226^d^
For the price^c^			3.72	3.70	3.84	3.74	3.77	3.86	0.250^d^
That are healthier^c^			4.15	4.11	4.11	4.09	4.16	4.09	0.874^d^
That make me cheerful, relaxed, active/awake^c^			3.65	3.57	3.64	3.65	3.65	3.77	0.320^d^
Natural, without additives or artificial/industrialized ingredients^c^			3.68	3.64	3.72	3.62	3.67	3.64	0.885^d^
Low in calories, sugars or fats^c^			3.63	3.66	3.65	3.59	3.63	3.63	0.978^d^
From the brand I always usually buy^c^			3.73	3.76	3.72	3.68	3.64	3.77	0.580^d^
Or foods similar to what I ate in childhood^c^			2.90	3.01	3.08	2.99	2.99	3.07	0.410^d^
That do not harm the environment, giving preference to organic foods and avoiding foods with pesticides^c^			3.59	3.75	3.69	3.66	3.61	3.61	0.397^d^

a P-values from Pearson's chi-square test.

b Mnimum wage value: R$ 998.00 (nine hundred and ninety-eight reais)/$229.42 (two hundred and twenty-nine dollars and forty-two cents).

c mean importance of factors related to food choice: 1 – “not at all important” to 5 – “very important.”

d ANOVA p-values.

### Understanding the nutritional content

In relation to the mean percentage of correct answers of the participants for the set of nine products, all FOPNL models performed significantly better than the CG (Table 3). In the presence of the octagon, circle and triangle, the percentages of correct answers were significantly higher than the percentage observed in the presence of the traffic light. The mean percentage of correct answers in the presence of the magnifier was similar to the percentage observed in the presence of the other four FOPNL models. In the analysis of each product individually, the mean percentage of correct answers in the presence of the octagon, magnifier, circle and triangle was higher than that of the CG for the nine products (Table 3). For the traffic light, the mean percentage of correct answers was significantly higher than for the GC for eight of the nine products.

**Table 3:** Performance of five models of front-of-package nutritional labeling in relation to the understanding of nutritional content, perception of healthiness and purchase intention. Brazil, 2019.

	Magnifier n = 400	Circle n = 400	Octagon n = 400	Traffic light n = 400	Triangle n = 400	Control n = 400	p
Understanding the nutritional content							
*Average percentage of correct answers for the set of nine products*^a^	59.5%^b^^c^	61.8%^c^	62.4%^c^	55.0%^b^	61.9%^c^	41.3%^a^	< 0,001
Requeijão^b^	64.6%^b^	68.6%^b^	67.5%^b^	63.8%^b^	66.6%^b^	51.7%^a^	< 0,001
Tomato sauce^b^	72.6%^b^	75.5%^b^	77.1%^b^	70.2%^b^	75.3%^b^	56.7%^a^	< 0,001
Sliced bread^b^	61.0%^c^	61.0%^c^	61.1%^c^	52.1%^b^	60.0%^b^,^c^	33.5%^a^	< 0,001
Cream cracker^b^	50.6%^b^	54.0%^c^	54.5%^c^	46.3%^b^	52.7%^b^,^c^	29.0%^a^	< 0,001
Cereal bar^b^	46.7%^b^	46.6%^b^	47.6%^b^	45.5%^b^	47.3%^b^	29.1%^a^	< 0,001
Morning cereal^b^	39.1%^b^	42.0%^b^	43.7%^b^	38.6%^b^	42.0%^b^	30.3%^a^	< 0,001
Nectar (juice)^b^	70.2%^b^	72.0%^b^	73.9%^b^	70.7%^b^	68.4%^b^	50.4%^a^	< 0,001
Corn^b^	72.7%^b^	74.2%^b^	75.0%^b^	62.6%^a^	75.1%^b^	55.9%^a^	< 0,001
Dinner roll^b^	58.8%^c^	63.2%^c^	62.9%^c^	49.4%^b^	62.8%^c^	37.2%^a^	< 0,001
Perception of healthiness							
*Mean perception of healthiness for the set of nine products*^c^	3.09^b^	2.91^b^	2.90^b^	3.09^b^	2.94^b^	3.40^a^	0.001
Requeijão^d^	3.00^a^,^b^	2.73^b^	2.71^b^	2.85^b^	2.84^b^	3.26^a^	0.001
Tomato sauce^d^	2.98^a^,^b^,^c^	2.84^c^	2.79^c^	3.17^a^,^b^	2.89^b^,^c^	3.25^a^	0.001
Sliced bread^d^	3.49^b^	3.34^b^	3.38^b^	3.64^a^	3.33^b^	3.89^a^	0.001
Cream cracker^d^	3.03^b^	2.78^b^	2.85^b^	2.89^b^	2.81^b^	3.47^a^	0.002
Cereal bar^d^	3.24^b^	3.05^b^	3.10^b^	3.09^b^	3.03^b^	3.66^a^	0.001
Morning cereal^d^	3.06^a^,^b^	2.81^b^	2.80^b^	2.88^b^	2.83^b^	3.34^a^	0.001
Nectar (juice)^d^	3.03^a^,^b^	2.87^b^	2.80^b^	3.11^a^,^b^	2.83^b^	3.30^a^	0.001
Corn^d^	3.08^a^,^b^,^c^	2.94^b^,^c^	2.96^b^,^c^	3.35^a^	3.04^b^,^c^	3.38^a^	0.001
Dinner roll^d^	2.94^a^,^b^	2.90^a^,^b^	2.76^b^	2.92^a^,^b^	2.91^a^,^b^	3.15^a^	0.001
Purchase intention							
*Average purchase intention for the set of nine products*^e^	3.39^b^	3.28^b^	3.26^b^	3.42^b^	3.28^b^	3.76^a^	0.001
Requeijão^f^	3.45^b^	3.26^b^	3.28^b^	3.35^b^	3.26^b^	3.80^a^	0.001
Tomato sauce^f^	3.53^a^,^b^,^c^	3.32^c^	3.39^b^,^c^	3.68^a^,^b^	3.37^b^,^c^	3.81^a^	0.001
Sliced bread^f^	3.60^b^	3.55^b^	3.57^b^	3.80^a^	3.51^b^	3.97^a^	0.001
Cream cracker^f^	3.28^b^	3.07^b^	3.19^b^	3.22^b^	3.14^b^	3.75^a^	0.001
Cereal bar^f^	3.36^b^	3.32^b^	3.26^b^	3.27^b^	3.26^b^	3.84^a^	0.001
Morning cereal^f^	3.29^b^	3.19^b^	3.09^b^	3.17^b^	3.18^b^	3.66^a^	0.001
Nectar^f^	3.25^a^	3.10^b^	3.03^b^	3.35^a^	3.11^b^	3.57^a^	0.001
Corn^f^	3.45^b^,^c^	3.39^b^,^c^	3.33^c^	3.72^a^,^b^	3.41^b^,^c^	3.83^a^	0.001
Dinner rolls^f^	3.36^a^	3.37^a^	3.25^b^	3.29^b^	3.30^b^	3.66^a^	0.001

Notes: ANOVA p-values. Equal lowercase letters on the same line indicate that the means are similar according to the Tukey test (p < 0.05).

a mean percentage of correct answers for the nine products (0 to 100%).

b mean percentage of correct answers for each product.

c Mean of the participants' perception of healthiness for the set of nine products: 1 – “not healthy to 5 – very healthy”.

d mean of participants' perception of healthiness for each product individually.

e Mean purchase intention of the participants for the set of nine products: 1 – “I would certainly not buy to 5 – I would certainly buy”

f Mean participants' purchase intent for each of the products.

### Perception of Healthiness

The performance of the five FOPNL models was significantly higher than the CG, reducing the means of perception of healthiness for all nine products (Table 3). In the analysis of the means of perception of healthiness for each product alone, the presence of the octagon was the only one that significantly reduced the perception of healthiness of the participants for all nine products, compared with the CG. The traffic light showed means lower than the GC for four products, and the magnifier, only for three products.

**Table 4:** Participants' perception of five models of front-of-package nutritional labeling. Brazil, 2019.

	Magnifier n = 400	Circle n = 400	Octagon n = 400	Traffic light n = 400	Triangle n = 400	Control n = 400	p
Perception of models							
I saw the label^a^	77.0%^c^,^b^	79.0%^b^,^a^	73.3%^c^	83.3%^a^	77.3%^c^,^b^	NA	0.015^b^
I found the nutritional information more quickly with this label^c^	4.19^a^	4.20^a^	4.09^a^	4.17^a^	4.18^a^	NA	0.710^d^
I understood this label^c^	4.49^a^,^b^	4.55^a^,^b^	4.59^b^	4.41^a^	4.57^a^,^b^	NA	0.035^d^
I understood the nutritional information more quickly with this label^c^	4.35^a^	4.37^a^	4.31^a^	4.35^a^	4.37^a^	NA	0.919^d^
I trusted the label^c^	4.21^a^	4.24^a^	4.09^a^	4.10^a^	4.24^a^	NA	0.109^d^
I would like to find this label on food packages^c^	4.66^a^	4.64^a^	4.62^a^	4.66^a^	4.70^a^	NA	0.706^d^
It was difficult to see the label^c^	2.4^a^,^b^	2.21^a^	2.58^b^	2.50^a^,^b^	2.38^a^,^b^	NA	0.009^d^
I felt uncomfortable with this label^c^	2.42^a^	2.38^a^	2.48^a^	2.35^a^	2.34^a^	NA	0.724^d^
The presence of this label made me afraid^c^	2.58^a^,^b^	2.69^a^,^b^	2.66^a^,^b^	2.34^a^	2.75^b^	NA	0.001^d^

NA not applicable.

Note: Equal lowercase letters on the same line indicate that the averages are similar according to the Tukey test (p < 0.05).

a Percentage of participants who answered yes to the question.

b Pearson chi-square test p-values.

c Participants' mean agreement on the acceptability of FOPNL models: “1 – totally disagree to 5 – totally agree”

d ANOVA p-values.

### Purchase intention

The presence of FOPNL reduced the purchase intention in relation to the CG for the product group investigated (Table 3), regardless of the FOPNL model. In the analysis of the means of purchase intention for each product individually, the octagon and triangle showed significantly lower means than the GC for the nine products. The circle showed lower averages than the CG for eight products. The magnifier and the traffic light showed means lower than the CG for only five of the nine products investigated.

### Consumer perception of FOPNL models

There was a significant difference between the five FOPNL models for the “I saw the label” items. The percentage of participants who declare to have seen the FOPNL ranged from 73.3% to 83.3%, being higher for the traffic light (83.3%) and circle (79.0%) than for the octagon (73.3%). The agreement for the item “I understood this label” was higher for the octagon (4.59) when compared with the traffic light (4.41).

Consumers supported the presence of FOPNL, perceiving it as reliable to increase the understanding of nutritional information. There was a high degree of agreement (means greater than 4, on a 5-point scale) for all positive items of perception of the models. For negative items, such as discomfort or difficulty in identifying the model, low agreement was observed (means less than 2.5). Despite the low agreement, the mean of the octagon (2.58) for the item “it was difficult to see the label” was higher than the mean of the circle (2.21). For the item “the presence of this label made me afraid,” the mean of the traffic light (2.34) was lower than that observed for the triangle (2.75).

## DISCUSSION

Understanding nutritional content is considered crucial to evaluate the effectiveness of nutritional labeling^4^^,^^5^. Warning FOPNL models (octagon, triangle, circle) performed better than the traffic light regarding the understanding of nutritional content. The superiority of warnings over traffic lights had already been reported in previous studies^6^^,^^10^^,^^21^^,^^27^.

Different from what had been reported by a previous study^9^, the magnifier model had a performance similar to the traffic light for this issue, and several factors may explain these results. Information processing, familiarity with the symbol used, the ability of the model to capture the consumer's attention and its color are factors already evidenced as important influencers to understand nutritional content^9^^,^^27^^,^^28^.

Regarding the processing of information, the traffic light does not show the same objectivity as the warnings, which only inform the nutrients present in high content in the product. Thus, a product may have, for example, a red (high) and two green labels (low), which may increase the perception of its healthiness, being this a possible limitation of the model^6^^,^^10^.

Regarding familiarity, the magnifier is the only model that is not widely used or standardized, being less familiar to the consumer than the warnings^9^. Familiarity with the symbol used in the FOPNL is essential to establish a fast and clear communication, enabling better understanding. According to the human information processing model, the internationally standardized and familiar warning signs are the triangle (sign most associated with risk), the octagon (associated with traffic stop sign), the traffic light and the red circle (used in traffic)^28^.

Understanding nutritional content is also related to attention capture, measured by the time necessary for the consumer to locate and visualize the FOPNL and the time required to identify the nutrients present in excess^27^. The traffic light and magnifier are FOPNL models that require longer attention capture time compared with warnings^9^. Warning models show images that repeat with each nutrient present in excess, drawing more consumer attention when compared with single-image models^29^.

Attention capture is also influenced by color, image or text presentation, position and symbol used in FOPNL^27^^–^^29^. The better performance of the warnings (octagon, triangle and circle) in relation to the traffic light, for understanding, may be related to color, since black captures attention faster, followed by red^27^.

All FOPNL models reduced the perception of product healthiness and purchase intention when compared with the CG, however, in the individual analysis, the traffic light and the magnifier reduced the perception of healthiness and purchase intention of a smaller number of products compared with the warnings. By perceiving a product as unhealthy, the consumer is expected to reduce the purchase intention^8^. One of the explanations for the lower performance of the traffic light in these two questions may be the presence of “low content” information in green color, which is usually associated with positive references and may increase perception of healthiness of the product even with the presence of high content of another critical nutrient, and consequently increase the purchase intention^6^^,^^9^^,^^10^. The lower performance of the magnifier may be related to the fact that it is the only model that does not have a design familiar to the consumer, requiring more effort to interpret the judgment regarding the healthiness of the food and the purchase decision, besides not being a model associated with risk, such as warnings, which have already been able to reduce the purchase intention in previous studies^9^^,^^17^^,^^21^^,^^26^.

The participants' perception was favorable to the presence of FOPNL in food packaging, understanding the FOPNL as reliable and easy to visualize and interpret to improve the understanding of the nutritional content of product, as already observed in similar studies^9^^,^^10^^,^^30^.

The percentage of participants who reported seeing the traffic light and the circle was higher than that observed for the octagon. The mean agreement for the item “it was difficult to see the label” was also higher for the octagon compared with the circle. These subjective findings, measured from the participants' perception, differ from a previous study, in which the difficulty of visualization was measured objectively, by time required to see the FOPNL with the use of software, when the circle required more time compared with the traffic light and the octagon^9^. We expected that the model that is easier to visualize also has better performance regarding the understanding of nutritional content, however, the octagon was the warning model that showed the highest percentage of correct answers (62.4%) for understanding nutritional content. The contradiction between results obtained by objective and subjective measures was reported by a previous study, suggesting that consumer perception may not accurately reflect the performance of FOPNL models^9^.

Regarding the item “the presence of this label made me afraid,” caution is needed in the interpretation of the results. Studies suggest limitations of the approach to basic emotions in detecting different aspects of emotional experience^26^. The higher mean of this item for the triangle compared with the traffic light may, for example, be in line with psychology studies that report that fear is an emotion activated by potentially threatening situations or real dangers^31^. In addition, it may also be aligned with the human communication model, which reports that the triangle is the most risk-associated sign^27^^,^^28^. The warnings (octagon, circle and triangle) were similar in this respect, corroborating the human communication model that reports that they are more familiar warning signs to consumers, and are also more commonly associated with risk^26^. We suggest that other investigations deepen the study of emotions associated with the presence of FOPNL, not restricted to fear, evaluating in detail its factors and the generated behaviors^31^.

Regarding the limitations of this study, we suggest that future research include the simulation of factors present in the actual purchasing situation, such as limited time, presence of nutritional claims and advertising on the food label, as well as a greater number of products, including healthy and unhealthy products.

This study was conducted with a robust and diverse sample in terms of sex, age group, education, income and region of the country. The control of these variables ensured homogeneity between the groups, which did not show statistical difference. Finally, it should be noted that the importance of factors related to food choice, such as price, ease of preparation, proximity to the place of purchase, preference for healthier foods, were similar between the exposure and control groups, and therefore were not factors influencing the performance of the FOPNL models in the studied sample.

## CONCLUSION

This study showed that FOPNL increases the understanding of nutritional content, reduces the perception of healthiness and the intention to buy foods with a high content of sugars, saturated fats and sodium. Warning FOPNL models (octagon, triangle and circle) showed superior performance to the traffic light for understanding. The magnifier model showed less consistent results than the warning models (octagon, triangle and circle). Regarding perception, the results revealed that consumers are favorable to the presence of FOPNL in food packages.

The results of this study bring important subsidies to public policy makers, reinforcing the need and advantages of the adoption of FOPNL in Brazil. Such a measure is urgent in a scenario where studies already point to rising prices of healthy food and cheapening of ultra-processed products in the coming years^32^. The choice of the FOPNL model to be adopted in the country should be discerning and consider the available scientific evidence, seeking to choose the model with the greatest potential for good performance, aligned with the particularities of the population that will benefit from it.
