# Supplementary material for: Performance and perception on front-of-package nutritional labeling models in Brazil
Source: Rev Saude Publica. 2021 Apr 23;55:19. doi: 10.11606/s1518-8787.2021055002395 (PMC8064653; doi:10.11606/s1518-8787.2021055002395)
Supplement: Translation

# Translation: Desempenho e percepção sobre modelos de rotulagem nutricional frontal no Brasil

**Authors:** Luisete Moraes Bandeira, Jéssica Pedroso, Natacha Toral, Muriel Bauermann Gubert

**Affiliations:** I BrasíliaDF Brasil Universidade de Brasília. Faculdade de Ciências da Saúde. Programa de Pós-Graduação em Saúde Coletiva. Asa Norte, Campus Universitário Darcy Ribeiro, Brasília, DF, Brasil; II BrasíliaDF Brasil Faculdade de Ciências da Saúde. Programa de Pós-Graduação em Nutrição Humana. Brasília, DF, Brasil

**Keywords:** Rotulagem Nutricional Frontal, Rotulagem Nutricional, Rotulagem de Alimentos, Aviso, Política Nutricional, Promoção da Saúde

## Abstract

**OBJETIVO:**

Avaliar o desempenho e a percepção de cinco modelos de rotulagem nutricional frontal (RNF) entre consumidores brasileiros.

**MÉTODOS:**

Estudo transversal, com aplicação de questionário online a 2.400 indivíduos, alocados de forma aleatória em seis grupos de estudo, sendo um controle e cinco expostos a modelos de RNF (octógono, triângulo, círculo, lupa e semáforo), aplicados a nove alimentos. Foi avaliado o entendimento do conteúdo nutricional, a percepção de saudabilidade, a intenção de compra e a percepção dos consumidores brasileiros sobre os modelos.

**RESULTADOS:**

Todos os modelos de RNF aumentaram o entendimento do conteúdo nutricional e reduziram a percepção de saudabilidade e a intenção de compra, quando comparados ao grupo controle (41,3%). Os modelos de RNF de advertência - octógono (62,4%), triângulo (61,9%) e círculo (61,8%) - apresentaram desempenho significativamente superior ao semáforo (55,0%) quanto ao entendimento do conteúdo nutricional. O desempenho do modelo da lupa (59,5%) não diferiu dos outros quatro modelos testados, inclusive do semáforo (55,0%), para o entendimento do conteúdo nutricional. A análise individual dos alimentos sugere melhor desempenho das advertências em relação à lupa e ao semáforo para a percepção de saudabilidade e a intenção de compra. Os consumidores manifestam-se favoráveis à presença da RNF, percebendo-a como confiável para aumentar o entendimento das informações nutricionais.

**CONCLUSÃO:**

A RNF deve ser implementada nos rótulos dos alimentos no Brasil, considerando que aumenta o entendimento nutricional, reduz a percepção de saudabilidade e a intenção de compra de alimentos com nutrientes críticos. As advertências apresentaram melhor desempenho quando comparadas aos demais modelos.

## INTRODUÇÃO

A rotulagem nutricional frontal (RNF) é recomendada internacionalmente^1^ como ferramenta para auxiliar o consumidor na interpretação das declarações quantitativas de nutrientes dos alimentos, geralmente de difícil entendimento e dispostas em letras pequenas no verso das embalagens^2^. Quase metade da população brasileira têm dificuldade de interpretar a informação nutricional dos rótulos dos alimentos^3^. Ao não compreender o conteúdo do alimento, o julgamento quanto à saudabilidade e, consequente, a decisão de compra do indivíduo são afetados^4^^,^^5^.

Vários países adotam diferentes modelos de RNF para ajudar o consumidor nessa interpretação. Os modelos de advertência (octógono, círculo e triângulo), informam, de maneira simples e direta, se o alimento apresenta alto teor de algum nutriente (açúcares, gorduras, sódio). Eles têm se mostrado mais eficientes em aumentar o entendimento, e consequentemente, reduzir a percepção de saudabilidade e a intenção de compra dos alimentos, quando comparados ao semáforo nutricional, que informa o baixo, médio e alto conteúdo dos nutrientes, ou ao *Guideline Daily Amounts* (GDA), que indica o percentual de nutriente presente no alimento em relação ao valor diário recomendado^6^^–^^10^. Nos últimos anos, quatro países da América Latina - Chile, Peru, Uruguai e México - adotaram a RNF de advertência no formato de octógono como obrigatória^11^^–^^14^.

No Brasil, a Agência Nacional de Vigilância Sanitária (Anvisa) aprovou, em 2020, um modelo de RNF em formato retangular preto com lupa, semelhante ao que vem sendo discutido no Canadá^15^^,^^16^. Contudo, apenas dois estudos avaliaram o desempenho deste modelo de RNF, sendo ele inferior ao octógono e ao triângulo em reduzir o tempo para identificação de nutrientes presentes em excesso, entre adultos brasileiros^9^. O modelo da lupa também se mostrou inferior ao octógono, círculo e triângulo em aumentar o entendimento do conteúdo nutricional entre adultos dos Estados Unidos, Canadá, Austrália e Reino Unido^17^.

O desempenho dos modelos de RNF pode, ainda, ser influenciado por fatores como a motivação para a saúde, a facilidade de preparo e o preço^18^, bem como por aspectos relacionados ao próprio design do modelo, como sua capacidade e chamar atenção, a facilidade do consumidor em identificar e processar sua informação, a familiaridade com a RNF e a percepção de risco por ela gerada^8^^,^^19^^–^^21^.

Em virtude disso, é importante a realização de estudos locais para identificar o modelo de RNF mais adequado para a população de cada país^22^. Observa-se a necessidade de estudos que comparem o desempenho de diferentes modelos de RNF no Brasil, incluindo a lupa, investigada em apenas um dos dois estudos realizados com adultos brasileiros que compararam mais de um modelo de RNF^9^^,^^10^.

Diante disso, o objetivo deste estudo foi avaliar o desempenho de cinco modelos de rotulagem nutricional frontal (octógono, triângulo, círculo, lupa e semáforo) em aumentar o entendimento do conteúdo nutricional, reduzir a percepção de saudabilidade e a intenção de compra de alimentos, além de identificar a percepção de consumidores adultos brasileiros sobre estes modelos e a importância de fatores relacionados à escolha alimentar.

## MÉTODOS

### Participantes do Estudo

Foi realizado estudo transversal com uma amostra de 2.400 indivíduos alocados de forma aleatória em seis grupos de estudo. A amostra foi feita por cotas, sendo representativa da população brasileira em relação a sexo, classe econômica e às cinco macrorregiões do país. O recrutamento dos participantes foi realizado de forma digital, por uma empresa especializada em pesquisas online que possui cadastro dos respondentes. Os convites foram enviados apenas para pessoas que atendiam ao perfil de cotas pré-determinado na amostra. Ç medida que as cotas eram finalizadas, os convites eram enviados para as demais cotas.

O questionário foi aplicado em agosto de 2019. Todos os indivíduos assentiram em sua participação por meio do Termo de Consentimento Livre e Esclarecido. O estudo foi aprovado pelo Comitê de Ética em Pesquisa com Seres Humanos da Faculdade de Ciências da Saúde da Universidade de Brasília (protocolo 67420817.7.0000.0030).

### Alocação da Amostra nos Grupos de Pesquisa

Os participantes foram alocados de forma aleatória em seis grupos, sendo 400 no grupo controle (CG) e os demais em um dos cinco grupos de exposição: 1) lupa (n = 400); 2) círculo (n = 400); 3) octógono (n = 400), 4) triângulo (n = 400) e 5) e semáforo nutricional (n = 400) (Figura 1). A inclusão de um grupo controle permitiu a comparação do desempenho dos modelos de RNF entre si e o desempenho de cada um individualmente em relação à ausência de RNF no alimento.

**Figura:** Modelos de rotulagem nutricional frontal testados e exemplos das imagens dos alimentos visualizadas pelos participantes. Brasil, 2019.

### Posição e Tamanho dos Modelos de RNF

Os modelos de RNF (Figura 1) foram aplicados no canto superior direito, em diferentes percentuais da área do painel principal do alimento: 15% se o alimento tinha alto conteúdo de açúcares, sódio e gordura saturada; 10% quando alto em dois destes nutrientes; e 5% em um destes nutrientes. Para a lupa foi sempre utilizada 10% da área.

A lupa, o octógono e o triângulo foram apresentados na cor preta e o círculo na cor vermelha. O semáforo nutricional foi apresentado nas cores vermelha, amarela e verde, apontando respectivamente os teores de alto, médio ou baixo dos nutrientes.

Para a definição do baixo, médio e alto teor de nutrientes (açúcares livres, gordura saturada e sódio) foi adotado o modelo de perfil nutricional mais restritivo proposto pela Anvisa^23^.

### Seleção dos Alimentos

Um painel de especialistas selecionou nove alimentos comumente consumidos pela população brasileira^24^ e usualmente percebidos como saudáveis, apesar de apresentarem alto conteúdo de, pelo menos, um nutriente (açúcares livres, gordura e sódio) (Tabela 1). Conforme realizado em estudo anterior^9^, as imagens dos alimentos foram elaboradas por empresa especializada em design gráfico exclusivamente para esta pesquisa e não continham alegações de saúde, marcas ou nomes comerciais, buscando neutralizar a influência destes fatores no desempenho dos modelos (Figura 1).

**Tabela 1:** Composição nutricional dos produtos incluídos no estudo e teor (baixo, médio e alto) dos nutrientes associados às doenças crônicas não transmissíveis, de acordo com os critérios estabelecidos no relatório preliminar de análise de impacto regulatório sobre rotulagem nutricional. Gerência Geral de Alimentos. Agência Nacional de Vigilância Sanitária, 2018.

Alimento	Tamanho da porção (g/mL)	Conteúdo de nutrientes por porção				Classificação do conteúdo de nutriente		
		Calorias (Kcal)	Açúcares livres (g)	Gorduras saturadas (g)	Sódio (mg)	Açúcares livres	Gorduras saturadas	Sódio
Requeijão	30	81	7	4,1	144	Médio	Alto	Alto
Molho de tomate	60	30	5,8	0	311	Baixo	Baixo	Alto
Pão de forma	50	133	20	0,5	219	Baixo	Baixo	Alto
Biscoito água e sal	30	129	20	1,7	210	Alto	Alto	Alto
Barra de cereal	22	91	16	1,6	17	Alto	Alto	Baixo
Cereal matinal	30	111	25	0	147	Alto	Baixo	Alto
Néctar	200	40	10	0	0	Alto	Baixo	Baixo
Milho verde	130	129	25	0	606	Baixo	Baixo	Alto
Bisnaguinha	40	124	22	0,8	189	Médio	Médio	Alto

### Coleta de Dados

O questionário foi organizado em três seções: 1) características dos participantes, 2) desempenho dos modelos de RNF e 3) percepção dos consumidores em relação aos modelos de RNF.

Na seção 1, foram identificadas as características dos participantes (sexo, faixa etária, escolaridade, renda e região de moradia), incluindo a importância de fatores relacionados à escolha alimentar. Foram elaborados dez itens baseados no *Food Choice Questionnaire*^18^, para os quais os participantes avaliaram a importância com opções de respostas em escala Likert de 5 pontos, variando de: 1 – “nem um pouco importante” a 5 – “muito importante”. Os itens eram “*Eu escolho alimentos*”: a) mais fáceis de preparar; b) que o local de compra é próximo para mim; c) pelo preço; d) que são mais saudáveis; e) que me deixam alegre, relaxado, ativo/acordado; f) naturais, sem aditivos ou ingredientes artificiais/industrializados; g) com poucas calorias, açúcares ou gorduras; h) da marca que sempre costumo comprar; i) ou comidas parecidas com o que comia na infância; j) que não prejudiquem o meio ambiente, dando preferência aos alimentos orgânicos e evitando alimentos com agrotóxicos.

Na seção 2, foi avaliado o desempenho dos modelos de RNF em aumentar o entendimento do conteúdo nutricional e em reduzir a percepção de saudabilidade e a intenção de compra dos alimentos apresentados. Os indivíduos visualizavam, individualmente e em ordem aleatória, os nove alimentos com o modelo de RNF segundo seu grupo de randomização. Nenhum indivíduo foi exposto a mais de um tipo de RNF. Enquanto visualizavam cada alimento, os participantes responderam a três perguntas. A primeira pergunta mensurava o entendimento do conteúdo nutricional do alimento: “*na sua opinião, esse alimento contém nutrientes em níveis mais altos do que o recomendado para uma dieta saudável?*”. Para fins de padronização e comparação entre os modelos de RNF estudados, optou-se por manter apenas essa pergunta para o semáforo, uma vez que esse era o único modelo de RNF que permitia quantificar níveis médio e baixo de nutrientes^9^^,^^10^. As opções de resposta eram de múltipla escolha: “muito açúcar”, “muito sódio”, “muita gordura saturada” ou “não contém nenhum nutriente em muita quantidade”, sendo possível que o participante marcasse mais de uma opção de reposta. Outras duas perguntas mensuraram a intenção de compra e a percepção de saudabilidade dos produtos, com opções de resposta em escala Likert de 5 pontos: “*você compraria este alimento?*” (1 – “eu certamente não compraria” a 5 – “eu certamente compraria”); e “*você considera este alimento”*: (1 – “nada saudável” a 5 – “muito saudável”). O grupo controle visualizou os mesmos alimentos, porém, sem qualquer modelo de RNF. Caso desejassem, os sujeitos de todos os grupos podiam visualizar a tabela de informação nutricional e a lista de ingredientes de cada alimento ao clicar em um botão disposto logo abaixo da imagem do alimento.

Na seção 3, foi avaliada a percepção dos participantes em relação aos modelos de RNF em relação à “facilidade de identificação”, à “confiabilidade”, ao “processamento da informação” e à “preferência”. Essas dimensões foram baseadas na estrutura de aceitabilidade proposta por Nielsen^25^, já utilizada em estudos sobre RNF^19^^,^^20^. As perguntas eram apresentadas enquanto o participante visualizava o modelo de RNF do seu grupo de randomização, de forma isolada. Para avaliar a facilidade de identificação, o indivíduo respondeu às seguintes perguntas/afirmação: “1. *Você viu que havia esse selo no alimento que você avaliou?* (sim ou não); “2. *foi difícil visualizar esse selo no alimento*”, “3. *encontrei mais rapidamente as informações nutricionais com esse selo*”. Para “confiabilidade”, a afirmativa era “4. *confiei nas informações deste selo*”. Quando avaliado o processamento da informação, as seguintes afirmações foram apresentadas - “5. *entendi mais rapidamente as informações nutricionais com esse selo*”, - “6. *entendi esse selo*”, “7. *me senti desconfortável com esse selo*”. A seguinte afirmação permitiu avaliar a preferência: “8. *eu gostaria de encontrar esse selo nas embalagens de alimentos*”.

Ainda nessa seção foi investigado se os modelos de RNF induziram os participantes à emoção básica medo^25^ por meio da afirmação: “9. *a presença desse selo me causou medo*”. As questões 2 à 9 tinham opções de resposta em escala do tipo Likert de 5 pontos, variando de 1 – “discordo totalmente” a 5 – “concordo totalmente”. Esta seção foi apresentada apenas para os participantes dos grupos exposição.

### Análises Estatísticas

Para o cálculo do tamanho amostral foi considerado nível de confiança de 95%, erro máximo aceitável de 2 pontos percentuais, alfa de 0,05 e poder do teste de 95%. No cálculo da amostra foi considerada a média de acertos para entendimento do conteúdo nutricional de 57,6% para o semáforo e de 79,9% para o triângulo^10^ e a utilização do teste ANOVA *one-way*, estimando-se um tamanho de efeito de 11. Assim, este estudo deveria incluir pelo menos 210 adultos por grupo, aos quais foram adicionados 100% para cobertura de possível perda ou inconsistência de dados, estimando-se uma amostra de 2.400 indivíduos. O cálculo foi realizado pelo programa G*Power 3.1.9.*2*.

Para o entendimento da RNF, primeiramente foi calculado o percentual de acertos de itens para cada alimento de acordo com a resposta do participante em relação à presença ou à ausência do nutriente em excesso. Para o conjunto dos nove alimentos, considerou-se o percentual de acertos do participante em relação a todos os alimentos. Posteriormente, foram comparadas as médias do percentual de acertos dos grupos de exposição e grupo controle.

Foram calculadas também, para os seis grupos, as médias de intenção de compra e de percepção de saudabilidade dos participantes em relação aos nove alimentos em conjunto e individualmente. A visualização do modelo de RNF foi expressa em percentual. Também foram calculadas as médias de concordância, segundo a escala do tipo Likert, considerada como variável contínua, para as questões que avaliaram a percepção dos participantes em relação aos modelos de RNF.

Utilizou-se teste qui-quadrado de Pearson (variáveis categóricas) ou ANOVA *one-way* com pós teste *Tukey* (variáveis contínuas) para verificar se existiam diferenças entre os grupos quanto às características dos participantes, o desempenho dos modelos de RNF e a percepção dos participantes em relação aos modelos de RNF entre os grupos. Foi considerado o intervalo de confiança de 95%. Todas as análises foram conduzidas por meio do software *Statistical Package for the Social Sciences* (SPSS), versão 23.0.

## RESULTADOS

A maioria da amostra de 2.400 adultos tinha idade entre 18 e 34 anos (55,1%), 51,2% eram mulheres e 37,1% possuíam ensino médio completo. As características dos participantes não apresentaram diferença estatística entre os seis grupos de pesquisa.

A média de importância dos fatores relacionados à escolha alimentar (facilidade de preparo, proximidade do local de compra, preço, preferência por alimentos mais saudáveis, com conteúdo natural, para controle de peso e a preocupação ética na escolha do alimento) atribuída pelos participantes não diferiu entre os grupos controle e exposição (Tabela 2).

**Tabela 2:** Características sociodemográficas dos participantes do estudo e média de importância atribuída aos fatores relacionados à escolha alimentar. Brasil, 2019.

Características		Amostra total n = 2.400 (%)	Lupa n = 400 (%)	Círculo n = 400 (%)	Octógono n = 400 (%)	Semáforo n = 400 (%)	Triângulo n = 400 (%)	Controle n = 400 (%)	p
Sexo									
	Masculino	48,8	48,8	48,0	47,8	49,5	49,5	49,3	0,993^a^
	Feminino	51,2	51,3	52,0	52,3	50,5	50,5	50,8	
Faixa etária,									
	18–34	55,1	52,8	54,8	56,5	55,5	53,0	58,0	0,940^a^
	35–54	35,9	37,8	35,5	35,5	35,5	37,0	34,0	
	≥ 55	9,0	9,5	9,8	8,0	9,0	10,0	8,0	
Escolaridade, em anos									
	< 9	3,3	3,3	4,8	3,5	2,0	3,8	2,3	
	9 < 12	10,8	9,5	12,0	9,8	11,8	10,0	11,8	0,510^a^
	≥ 12	86,0	87,3	83,3	86,8	86,3	86,3	86,0	
Renda, em salários-mínimos^b^									
	< 2	48,4	45,8	47,5	48,8	49,0	49,8	49,5	
	2 < 10	46,1	48,3	46,0	48,3	45,3	43,8	45,3	0,569^a^
	≥ 10	5,5	6,0	6,5	3,0	5,8	6,5	5,3	
Região									
	Norte	5,1	4,5	5,25	5,5	4,75	5,25	5,75	
	Centro-Oeste	8,3	8,5	7,5	9	8,5	9	7,75	
	Nordeste	23	24,5	23	21,8	22,3	24,5	22,5	1,000^a^
	Sudeste	47,8	46,5	48	48,3	50,2	45,5	48,5	
	Sul	15,5	16	16,25	15,5	14,25	15,75	15,5	
Importância de fatores relacionados à escolha alimentar – “*eu escolho alimentos*:”									
Mais fáceis de preparar^c^			3,69	3,69	3,51	3,67	3,67	3,80	0,049^d^
Que o local de compra é próximo para mim^c^			3,86	3,66	3,83	3,80	3,83	3,77	0,226^d^
Pelo preço^c^			3,72	3,70	3,84	3,74	3,77	3,86	0,250^d^
Que são mais saudáveis^c^			4,15	4,11	4,11	4,09	4,16	4,09	0,874^d^
Que me deixam alegre, relaxado, ativo/acordado^c^			3,65	3,57	3,64	3,65	3,65	3,77	0,320^d^
Naturais, sem aditivos ou ingredientes artificiais/industrializados^c^			3,68	3,64	3,72	3,62	3,67	3,64	0,885^d^
Com poucas calorias, açúcares ou gorduras^c^			3,63	3,66	3,65	3,59	3,63	3,63	0,978^d^
Da marca que sempre costumo comprar^c^			3,73	3,76	3,72	3,68	3,64	3,77	0,580^d^
Ou comidas parecidas com o que comia na infância^c^			2,90	3,01	3,08	2,99	2,99	3,07	0,410^d^
Que não prejudiquem o meio ambiente, dando preferência aos alimentos orgânicos e evitando alimentos com agrotóxicos^c^			3,59	3,75	3,69	3,66	3,61	3,61	0,397^d^

a p-valores do teste qui-quadrado de Pearson.

b Valor do saláriomínimo: R$998,00 (novecentos e noventa e oito reais)/$229,42 (duzentos e vinte e nove dólares e quarenta e dois centavos).

c Média de importância de fatores relacionados à escolha alimentar: 1 - “nem um pouco importante a 5 - muito importante”

d p-valores da ANOVA.

### Entendimento do Conteúdo Nutricional

Em relação ao percentual médio de acertos dos participantes para o conjunto de nove alimentos, todos os modelos de RNF tiveram desempenho significativamente superior ao GC (Tabela 3). Na presença do octógono, círculo e triângulo, os percentuais de acerto foram significativamente superiores ao percentual observado na presença do semáforo. O percentual médio de acertos na presença da lupa não foi diferente do percentual observado na presença dos outros quatro modelos de RNF. Na análise de cada alimento individualmente, o percentual médio de acertos na presença do octógono, da lupa, do círculo e do triângulo foi superior ao do GC para os nove alimentos (Tabela 3). Para o semáforo, o percentual médio de acertos foi significativamente superior ao do CG para oito dos nove alimentos.

**Tabela 3:** Desempenho de cinco modelos de rotulagem nutricional frontal em relação ao entendimento do conteúdo nutricional, percepção de saudabilidade e intenção de compra. Brasil, 2019.

	Lupa n = 400	Círculo n = 400	Octógono n = 400	Semáforo n = 400	Triângulo n = 400	Controle n = 400	p
Entendimento do conteúdo nutricional							
*Percentual médio de acertos para o conjunto de nove produtos*^a^	59,5%^b^,^c^	61,8%^c^	62,4%^c^	55,0%^b^	61,9%^c^	41,3%^a^	< 0,001
Requeijão^b^	64,6%^b^	68,6%^b^	67,5%^b^	63,8%^b^	66,6%^b^	51,7%^a^	< 0,001
Molho de tomate^b^	72,6%^b^	75,5%^b^	77,1%^b^	70,2%^b^	75,3%^b^	56,7%^a^	< 0,001
Pão de forma^b^	61,0%^c^	61,0%^c^	61,1%^c^	52,1%^b^	60,0%^b^,^c^	33,5%^a^	< 0,001
Biscoito água e sal^b^	50,6%^b^	54,0%^c^	54,5%^c^	46,3%^b^	52,7%^b^,^c^	29,0%^a^	< 0,001
Barra de cereal^b^	46,7%^b^	46,6%^b^	47,6%^b^	45,5%^b^	47,3%^b^	29,1%^a^	< 0,001
Cereal matinal^b^	39,1%^b^	42,0%^b^	43,7%^b^	38,6%^b^	42,0%^b^	30,3%^a^	< 0,001
Néctar^b^	70,2%^b^	72,0%^b^	73,9%^b^	70,7%^b^	68,4%^b^	50,4%^a^	< 0,001
Milho verde^b^	72,7%^b^	74,2%^b^	75,0%^b^	62,6%^a^	75,1%^b^	55,9%^a^	< 0,001
Bisnaguinha^b^	58,8%^c^	63,2%^c^	62,9%^c^	49,4%^b^	62,8%^c^	37,2%^a^	< 0,001
Percepção de saudabilidade							
*Média de percepção de saudabilidade para o conjunto de nove produtos*^c^	3,09^b^	2,91^b^	2,90^b^	3,09^b^	2,94^b^	3,40^a^	0,001
Requeijão^d^	3,00^a^,^b^	2,73^b^	2,71^b^	2,85^b^	2,84^b^	3,26^a^	0,001
Molho de tomate^d^	2,98^a^,^b^,^c^	2,84^c^	2,79^c^	3,17^a^,^b^	2,89^b^,^c^	3,25^a^	0,001
Pão de forma^d^	3,49^b^	3,34^b^	3,38^b^	3,64^a^	3,33^b^	3,89^a^	0,001
Biscoito água e sal^d^	3,03^b^	2,78^b^	2,85^b^	2,89^b^	2,81^b^	3,47^a^	0,002
Barra de cereal^d^	3,24^b^	3,05^b^	3,10^b^	3,09^b^	3,03^b^	3,66^a^	0,001
Cereal matinal^d^	3,06^a^,^b^	2,81^b^	2,80^b^	2,88^b^	2,83^b^	3,34^a^	0,001
Néctar^d^	3,03^a^,^b^	2,87^b^	2,80^b^	3,11^a^,^b^	2,83^b^	3,30^a^	0,001
Milho verde^d^	3,08^a^,^b^,^c^	2,94^b^,^c^	2,96^b^,^c^	3,35^a^	3,04^b^,^c^	3,38^a^	0,001
Bisnaguinha^d^	2,94^a^,^b^	2,90^a^,^b^	2,76^b^	2,92^a^,^b^	2,91^a^,^b^	3,15^a^	0,001
Intenção de compra							
*Média de intenção de compra para o conjunto de nove produtos*^e^	3,39^b^	3,28^b^	3,26^b^	3,42^b^	3,28^b^	3,76^a^	0,001
Requeijão^f^	3,45^a^	3,26^b^	3,28^b^	3,35^b^	3,26^b^	3,80^a^	0,001
Molho de tomate^f^	3,53^a^,^b^,^c^	3,32^c^	3,39^b^,^c^	3,68^a^,^b^	3,37^b^,^c^	3,81^a^	0,001
Pão de forma^f^	3,60^b^	3,55^b^	3,57^b^	3,80^a^	3,51^b^	3,97^a^	0,001
Biscoito água e sal^f^	3,28^b^	3,07^b^	3,19^b^	3,22^b^	3,14^b^	3,75^a^	0,001
Barra de cereal^f^	3,36^b^	3,32^b^	3,26^b^	3,27^b^	3,26^b^	3,84^a^	0,001
Cereal matinal^f^	3,29^b^	3,19^b^	3,09^b^	3,17^b^	3,18^b^	3,66^a^	0,001
Néctar^f^	3,25^a^	3,10^b^	3,03^b^	3,35^a^	3,11^b^	3,57^a^	0,001
Milho verde^f^	3,45^b^,^c^	3,39^b^,^c^	3,33^c^	3,72^a^,^b^	3,41^b^,^c^	3,83^a^	0,001
Bisnaguinha^f^	3,36^a^	3,37^a^	3,25^b^	3,29^b^	3,30^b^	3,66^a^	0,001

Notas: p-valores da ANOVA. Letras minúsculas iguais na mesma linha indicam que as médias não diferem de acordo com o teste Tukey (p < 0,05).

a Percentual médio de acertos para os nove produtos (0 a 100%).

b Percentual médio de acertos para cada produto.

c Média da percepção de saudabilidade dos participantes para o conjunto de nove produtos: 1 - “nada saudável a 5 - muito saudável”.

d Média da percepção de saudabilidade dos participantes para cada produto individualmente.

e Média da intenção de compra dos participantes para o conjunto de nove produtos: 1 - “eu certamente não compraria a 5 eu certamente compraria”.

f Média da intenção de compra dos participantes para cada um dos produtos.

### Percepção de Saudabilidade

O desempenho dos cinco modelos de RNF foi significativamente superior ao GC, reduzindo as médias de percepção de saudabilidade para o conjunto dos nove alimentos (Tabela 3). Na análise das médias de percepção de saudabilidade para cada alimento isoladamente, a presença do octógono foi a única que reduziu significativamente a percepção de saudabilidade dos participantes para todos os nove alimentos, comparado ao GC. O semáforo apresentou médias inferiores ao GC para quatro alimentos e a lupa, apenas para três alimentos.

### Intenção de Compra

A presença de RNF reduziu a intenção de compra em relação ao GC para o conjunto alimentos investigados (Tabela 3), independente do modelo de RNF. Na análise das médias de intenção de compra para cada alimento individualmente, o octógono e triângulo apresentaram médias significativamente menores do que o GC para os nove alimentos. O círculo apresentou médias inferiores ao GC para oito alimentos. Já a lupa e o semáforo apresentaram médias inferiores ao GC apenas para cinco dos nove alimentos investigados.

### Percepção dos Consumidores sobre os Modelos de RNF

Houve diferença significativa entre os cinco modelos de RNF para os itens “visualizei o selo”. O percentual de participantes que declaram ter visualizado a RNF variou de 73,3% a 83,3%, sendo maior para o semáforo (83,3%) e círculo (79,0%) do que para o octógono (73,3%). A concordância para o item “entendi esse selo” foi maior para o octógono (4,59) quando comparado ao semáforo (4,41).

Os consumidores manifestaram-se favoráveis à presença da RNF, percebendo-a como confiável para aumentar o entendimento das informações nutricionais. Verificou-se alto grau de concordância (médias superiores a 4, em escala de 5 pontos) para todos os itens positivos de percepção dos modelos. Para os itens negativos, como desconforto ou dificuldade para identificação do modelo, observou-se baixa concordância (médias inferiores a 2,5). Apesar da baixa concordância, a média do octógono (2,58) para o item “foi difícil visualizar o selo” foi superior à média do círculo (2,21). Para o item “a presença desse selo me causou medo”, a média do semáforo (2,34) foi inferior à observada para o triângulo (2,75).

**Tabela 4:** Percepção dos participantes em relação a cinco modelos de rotulagem nutricional frontal. Brasil, 2019.

	Lupa n = 400	Círculo n = 400	Octógono n = 400	Semáforo n = 400	Triângulo n = 400	Controle n = 400	p
Percepção sobre os modelos							
Visualizei o selo^a^	77,0%^c^,^b^	79,0%^b^,^a^	73,3%^c^	83,3%^a^	77,3%^c^,^b^	NA	0,015^b^
Encontrei mais rapidamente as informações nutricionais com esse selo^c^	4,19^a^	4,20^a^	4,09^a^	4,17^a^	4,18^a^	NA	0,710^d^
Entendi esse selo^c^	4,49^a^,^b^	4,55^a^,^b^	4,59^b^	4,41^a^	4,57^a^,^b^	NA	0,035^d^
Entendi mais rapidamente as informações nutricionais com esse selo^c^	4,35^a^	4,37^a^	4,31^a^	4,35^a^	4,37^a^	NA	0,919^d^
Confiei no selo^c^	4,21^a^	4,24^a^	4,09^a^	4,10^a^	4,24^a^	NA	0,109^d^
Gostaria de encontrar esse selo nas embalagens de alimentos^c^	4,66^a^	4,64^a^	4,62^a^	4,66^a^	4,70^a^	NA	0,706^d^
Foi difícil visualizar o selo^c^	2,4^a^,^b^	2,21^a^	2,58^b^	2,50^a^,^b^	2,38^a^,^b^	NA	0,009^d^
Senti-me desconfortável com esse selo^c^	2,42^a^	2,38^a^	2,48^a^	2,35^a^	2,34^a^	NA	0,724^d^
A presença desse selo me causou medo^c^	2,58^a^,^b^	2,69^a^,^b^	2,66^a^,^b^	2,34^a^	2,75^b^	NA	0,001^d^

NA não se aplica.

Nota: Letras minúsculas iguais na mesma linha indicam que as médias não diferem de acordo com o teste Tukey (p < 0,05).

a Percentual de participantes que respondeu sim para a pergunta.

b p-valores do teste qui-quadrado de Pearson.

c Média de concordância dos participantes em relação a aceitabilidade dos modelos de RNF: “1 - discordo totalmente a 5 - concordo totalmente”.

d p-valores da ANOVA.

## DISCUSSÃO

O entendimento do conteúdo nutricional é considerado crucial para avaliar a efetividade da rotulagem nutricional^4^^,^^5^. Os modelos de RNF de advertências (octógono, triângulo, círculo) apresentaram desempenho superior ao semáforo no que se refere ao entendimento do conteúdo nutricional. A superioridade das advertências em relação ao semáforo já havia sido relatada em estudos anteriores^6^^,^^10^^,^^21^^,^^27^.

Diferente do que havia sido reportado por estudo anterior^9^, não se observou melhor desempenho do modelo da lupa em relação ao semáforo para este quesito e vários fatores podem explicar esses resultados. O processamento da informação, a familiaridade com o símbolo utilizado, a capacidade do modelo em capturar a atenção do consumidor e a sua cor são fatores já evidenciados como importantes influenciadores do entendimento do conteúdo nutricional^9^^,^^27^^,^^28^.

Em relação ao processamento da informação, o semáforo não apresenta a mesma objetividade das advertências, que informam apenas os nutrientes presentes em alto teor no alimento. Desta forma, um alimento pode ter, por exemplo, um campo vermelho (alto) e dois verdes (baixo), o que pode aumentar a percepção de saudabilidade do mesmo, sendo essa uma possível limitação do modelo^6^^,^^10^.

Já em relação à familiaridade, a lupa é o único modelo que não é amplamente utilizado ou padronizado, sendo menos familiar ao consumidor do que as advertências^9^. A familiaridade ao símbolo utilizado na RNF é essencial para se estabelecer uma comunicação rápida e clara, possibilitando melhor entendimento. De acordo com o modelo de processamento da informação humana, os sinais de alertas familiares e padronizados internacionalmente são o triângulo (sinal mais associado ao risco), o octógono (associado a placa de trânsito pare), o semáforo e o círculo vermelho (utilizados no trânsito)^28^.

O entendimento do conteúdo nutricional também está relacionado à captura de atenção, mensurada pelo tempo necessário para o consumidor localizar e visualizar a RNF e pelo tempo requerido para identificar os nutrientes presentes em excesso^27^. O semáforo e a lupa são modelos de RNF que exigem maior tempo de captura de atenção em comparação com as advertências^9^. Os modelos de advertência apresentam imagens que se repetem a cada nutriente presente em excesso, chamando mais atenção do consumidor quando comparados aos modelos com imagem única^29^.

A captura de atenção é também influenciada pela cor, apresentação em imagem ou texto, posição e símbolo utilizado na RNF^27^^–^^29^. O melhor desempenho das advertências (octógono, triângulo e círculo) em relação ao semáforo, para o entendimento, pode estar relacionado com a cor, já que o preto apresenta captura de atenção mais rápida, seguida do vermelho^27^.

Todos os modelos de RNF reduziram a percepção de saudabilidade dos alimentos e a intenção de compra se comparados ao GC, entretanto, na análise individual, o semáforo e a lupa reduziram a percepção de saudabilidade e intenção de compra de um número menor de alimentos em comparação às advertências. Ao perceber um alimento como não saudável, espera-se que o consumidor reduza a intenção de compra^8^. Uma das explicações para o desempenho inferior do semáforo nestes dois quesitos, pode ser a presença da informação “baixo conteúdo” na cor verde, cor normalmente associada a referências positivas, o que pode aumentar percepção de saudabilidade do alimento mesmo com a presença de alto teor de outro nutriente crítico e consequentemente aumentar a intenção de compra^6^^,^^9^^,^^10^. Já o desempenho inferior da lupa pode estar relacionado ao fato deste ser o único modelo que não tem design familiar ao consumidor, exigindo mais esforço de interpretação para o julgamento quanto à saudabilidade do alimento e a decisão de compra, além de não ser um modelo associado ao risco, como as advertências, as quais já se mostraram capazes de reduzir a intenção de compra em estudos anteriores ^9^^,^^17^^,^^21^^,^^26^.

A percepção dos participantes foi favorável à presença da RNF nas embalagens dos alimentos, entendendo a RNF como confiável e de fácil visualização e interpretação para melhorar o entendimento do conteúdo nutricional dos alimentos, conforme já observado em estudos semelhantes^9^^,^^10^^,^^30^.

O percentual de participantes que declarou ter visualizado o semáforo e o círculo foi superior ao observado para o octógono. A média de concordância para o item “foi difícil visualizar o selo” também foi maior para o octógono em comparação ao círculo. Estes achados subjetivos, mensurados a partir da percepção dos participantes, diferem de estudo anterior, no qual a dificuldade de visualização foi mensurada de forma objetiva, por meio do tempo necessário para visualizar a RNF com a utilização de software, quando o círculo exigiu mais tempo em comparação com o semáforo e o octógono^9^. É esperado que o modelo de mais fácil visualização também apresente melhor desempenho quanto ao entendimento do conteúdo nutricional, entretanto, o octógono foi o modelo de advertência que apresentou maior percentual de acertos (62,4%) para o entendimento do conteúdo nutricional. A contradição entre resultados obtidos por medidas objetivas e subjetivas foi reportada por estudo anterior, sugerindo que a percepção dos consumidores pode não refletir com precisão o desempenho dos modelos de RNF^9^.

Em relação ao item “a presença desse selo me causou medo” é preciso cautela na interpretação dos resultados. Estudos sugerem limitações da abordagem das emoções básicas em detectar diferentes aspectos da experiência emocional^26^. A maior média deste quesito para o triângulo comparado ao semáforo pode estar, por exemplo, alinhada aos estudos da psicologia que reportam que o medo é uma emoção ativada por situações potencialmente ameaçadoras ou por perigos reais^31^. Além disso, também pode estar alinhada ao modelo de comunicação humana, que reporta ser o triângulo o sinal mais associado à risco^27^^,^^28^. As advertências (octógono, círculo e triângulo) não diferiram entre si neste quesito, corroborando com o modelo da comunicação humana que reporta serem sinais de alerta mais familiares aos consumidores, sendo também mais comumente associados ao risco^26^. Sugere-se que outros estudos aprofundem o estudo das emoções associadas à presença a RNF, não se restringindo ao medo, avaliando com detalhes seus fatores e os comportamentos gerados^31^.

No que se refere às limitações deste estudo, sugere-se que pesquisas futuras incluam a simulação de fatores presentes na situação real de compra, tais como tempo limitado, presença de alegações nutricionais e publicidade no rótulo dos alimentos, além de número maior de alimentos, incluindo produtos saudáveis e não saudáveis.

O presente estudo foi realizado com uma amostra robusta e diversa em termos de sexo, faixa etária, escolaridade, renda e região do país. O controle das dessas variáveis garantiu homogeneidade entre os grupos, as quais não apresentaram diferença estatística. Por fim, cabe salientar que a importância de fatores relacionados à escolha alimentar como, por exemplo o preço, a facilidade de preparo, a proximidade do local de compra, a preferência por alimentos mais saudáveis, não diferiu entre os grupos de exposição e controle, não sendo, portanto, fatores influenciadores do desempenho dos modelos de RNF na amostra estudada.

## Conclusão

Este estudo evidenciou que a RNF aumenta o entendimento do conteúdo nutricional, reduz a percepção de saudabilidade e a intenção de compra de alimentos com alto conteúdo de açúcares, gorduras saturadas e sódio. Os modelos de RNF de advertências (octógono, triângulo e círculo) apresentaram desempenho superior ao semáforo para o entendimento. O modelo da lupa apresentou resultados menos consistentes do que os modelos de advertência (octógono, triângulo e círculo). Em relação à percepção, os resultados revelaram que os consumidores são favoráveis à presença da RNF nos alimentos.

Os resultados deste estudo trazem importantes subsídios aos formuladores de políticas púbicas, reforçando a necessidade e vantagens da adoção da RNF no Brasil. Tal medida é urgente em um cenário onde estudos já apontam aumento de preços dos alimentos saudáveis e barateamento de produtos ultraprocessados nos próximos anos^32^. A escolha do modelo de RNF a ser adotado no país deve ser criteriosa e considerar as evidências científicas disponíveis, buscando escolher o modelo com maior potencial de bom desempenho, alinhado às particularidades da população que dele se beneficiará.
